# The Composition of Koch’s Lymph

**Published:** 1891-03

**Authors:** 


### The Composition of Koch's Lymph.

Professor Koch has made a third report of his work under date of January 15. His reasons there given for withholding from the public to the present time the exact composition of the fluid used in the treatment of tuberculosis, should be satisfactory to the medical profession. The modest manner in which he now refers to his previous labors, and the methods employed by which he has reached his present conclusions commands approval, and the unreserved manner in which he now gives to the public the results of his labors must surely disarm those who were disposed to criticise the withholding of his discovery from an exacting and impatient multitude.

"The remedy," says Professor Koch, "which is used in the new treatment consists of a glycerine extract derived from the pure cultivation of tubercle bacilli."

This extract contains not only the effective substance derived from the bacilli, but such other substances, consisting of salts, coloring material, and extractive matters as would be soluble in a fifty per cent. solution of glycerine. Their elimination is of no practical importance since they exert no essential influence upon the human organism. Combined with other extractive material the effective matter is precipitated by alcohol, in which it is insoluble, and can be isolated from other substances in a comparatively pure and concentrated form and with increased potency.

Regarding the composition of the effective substance, as he terms it, Professor Koch says that for the present only surmises can be entertained. He believes it to be derivative from albuminous bodies, and having close affinity to them. That it does not belong to the group of tox-albumens he argues from the fact of its tolerance of high temperature and of its behavior in the dialyser. So far as can be estimated the percentage of the active principle in this solution is exceedingly small, being rated at a fraction of one per cent. It is evident that we here have to do with a remedy which, considering the effects which it produces, is far more potent than any drug hitherto employed.

Various views are entertained as to the manner in which the substance produces its effects.

It is known that tubercular bacilli, when growing in living tissues, produce substances which affect unfavorably, and in concentrated form, produce necrosis of living tissue. In this necrotic tissue the bacillus fails to obtain its needed nourishment, ceases its development and sometimes dies.

Thus the very environment of the bacillus is inimical to its development and multiplication. If now a substance be introduced that can develop the necrotic environment still further, and limit the possibilities of its growth, by so much the more will its limitation and destruction be assured.

It may be as yet impossible to explain the manner in which this remedy exerts its specific influence upon tuberculous tissue. Nor can we yet understand the remarkable rapidity with which its effects are produced. Nor does time yet suffice to determine as to the permanency of alleged favorable results. Instances are cited where the tubercular bacilli disappeared from the sputa of patients while under treatment, and at the end of three months had not reappeared, the patients in the mean time improving in health, the physical signs of phthisis having disappeared.

